# Atraumatic Isolated Dislocation of Pisiform With Ulnar Nerve Palsy

**DOI:** 10.7759/cureus.46042

**Published:** 2023-09-27

**Authors:** Carolyn Choong Yoke Lin, Sallehuddin Hassan

**Affiliations:** 1 Hand Unit, Orthopedics, and Traumatology, Hospital Sultanah Bahiyah, Alor Setar, MYS

**Keywords:** paediatric orthopedics, hand surgery, carpal bone dislocation, hand-wrist bones, pisiform bone

## Abstract

Isolated pisiform dislocation is an uncommon condition, with a limited number of cases reported in the literature. We present a unique case of a 15-year-old male who experienced an atraumatic isolated dislocation of the pisiform bone in his left wrist and presented with pain, deformity, and ulnar nerve palsy in his little and ring fingers. Radiographic investigations confirmed the diagnosis of isolated pisiform dislocation, and the patient successfully underwent an open reduction, stabilization of pisiform, and exploration of the ulnar nerve.

## Introduction

Pisiform is a sesamoid bone located within the flexor carpi ulnaris (FCU) tendon. Dislocation of the pisiform bone is an unusual and uncommon injury. Even rarer is its occurrence in the absence of other carpal bone involvement. To date, there have been several reported cases in literature of isolated pisiform dislocation due to trauma. We report a rare case of atraumatic isolated dislocation of pisiform in a teenager.

This article was previously presented as an oral presentation at the 21st Asia Pacific Orthopaedics Association (APOA) Congress, Virtual Conference on July 29-31, 2021.

## Case presentation

A 15-year-old boy presented with left wrist pain and deformity for two weeks duration, after waking up from sleep. He subsequently developed weakness and numbness over his left little and ring fingers. The patient denied a prior history of trauma to the affected wrist. He also denied any heavy lifting activity before the presentation.

**Figure 1:**
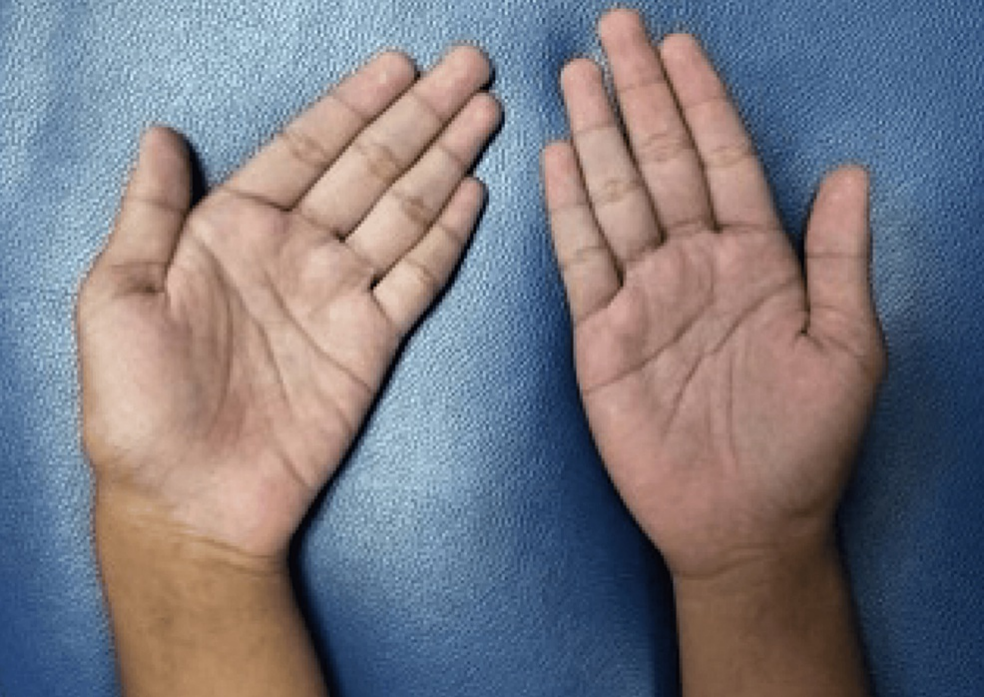
Left wrist kept in ulnar deviation as compared to the right.

Clinical examination revealed an ulnar-deviated left wrist (Figure 1), with point tenderness over the palmar aspect of the hypothenar eminence. The wrist motion was restricted due to pain. Reduced sensation was noted over the ulnar one and a half fingers. There was also a weak abduction of his left little finger. His Beighton score was 2, indicating no underlying ligament or joint hyperlaxity. Ulnar artery pulse was present. 

**Figure 2:**
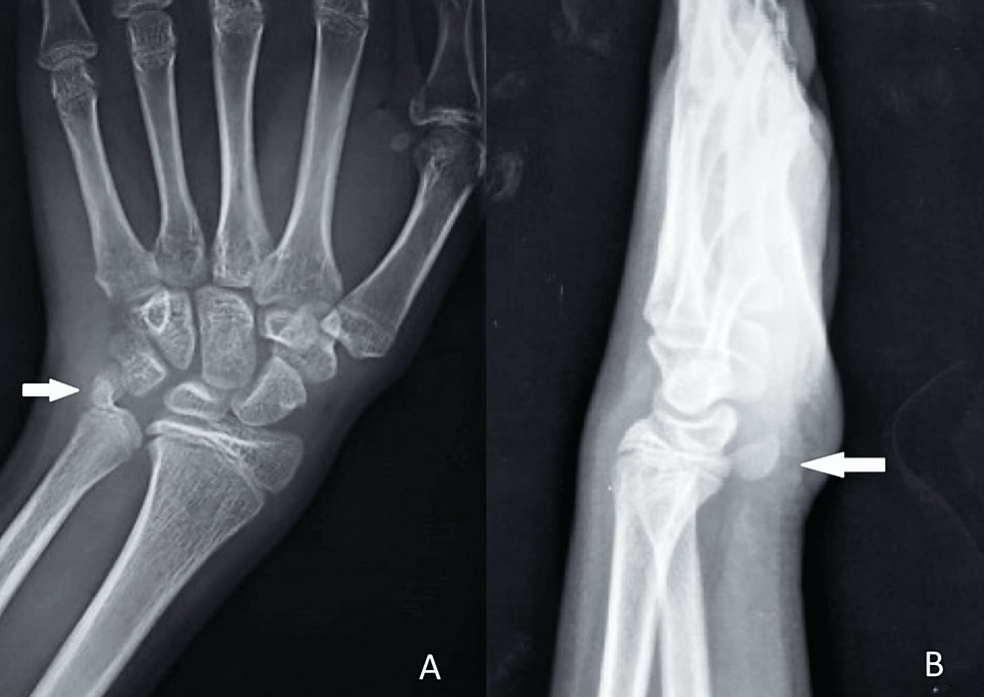
Plain radiographs showing the pisiform bone (white arrow) lying close to the ulnar styloid on (A) AP views and just anterior to the lunate on (B) lateral views. AP, anteroposterior

The plain radiographs of the left wrist showed an isolated dislocation of the pisiform bone. The pisiform bone was located proximally and medially toward the ulnar styloid on anteroposterior (AP) views and pulled volar, just proximal to the proximal border of the lunate on lateral views. The pisiform bone was no longer articulating with the triquetrium (Figure 2). 

**Figure 3:**
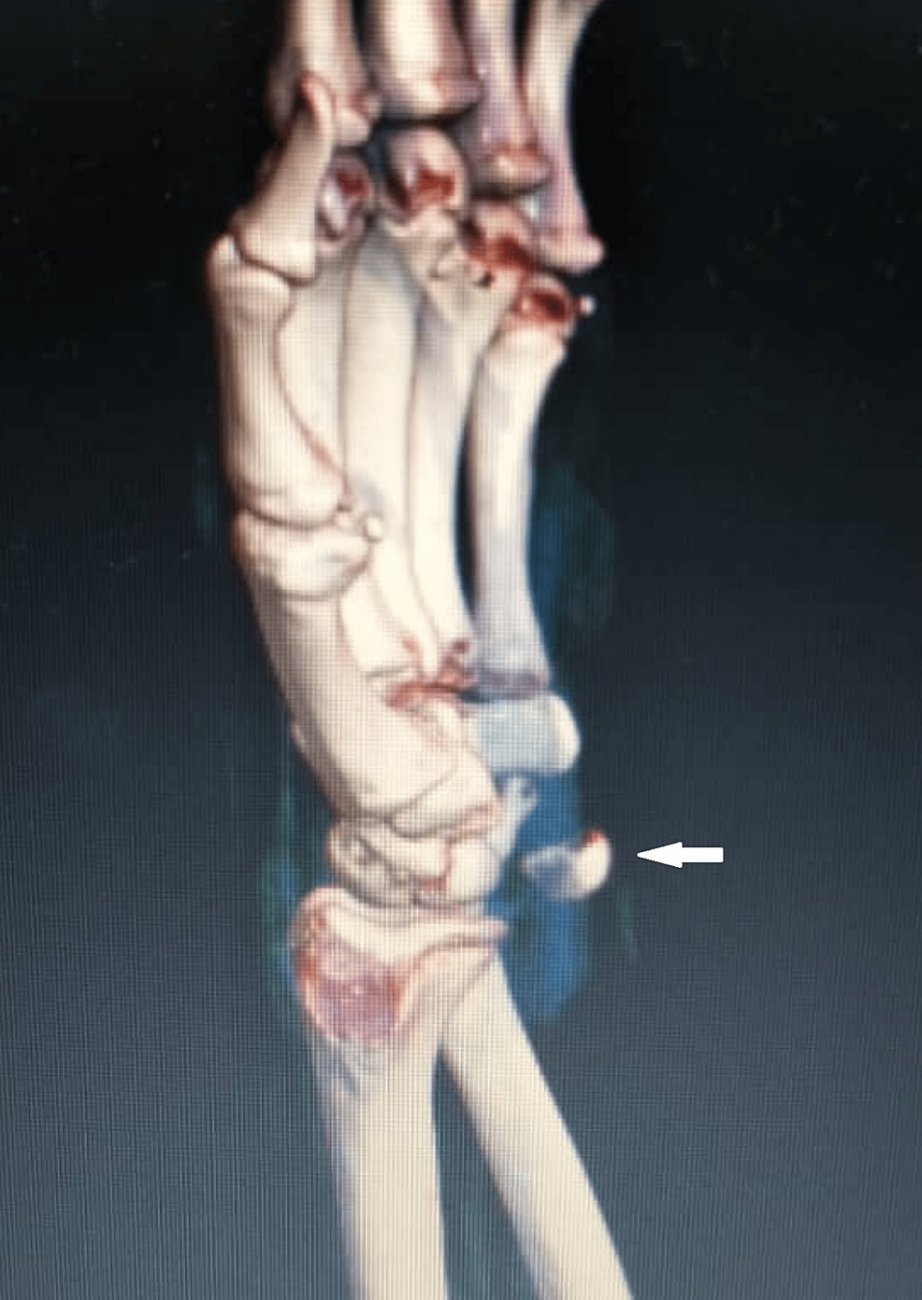
A three-dimensional computed tomography of the left wrist demonstrating the position of the dislocated pisiform bone (white arrow).

A computed tomographic scan (CT scan) of the left wrist showed no involvement of other carpal bones except for the dislocated pisiform (Figure 3).

**Figure 4:**
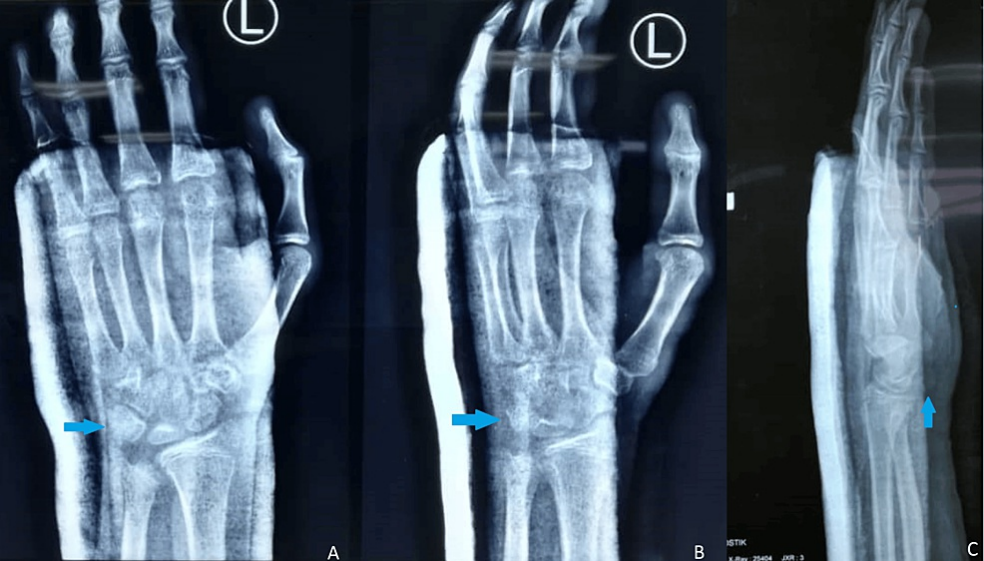
Postoperative plain radiographs showing the relocation of the pisiform bone (blue arrow) in (A) AP view, (B) oblique view, and (C) true lateral view. AP, anteroposterior

The patient underwent surgical exploration of the ulnar nerve and open reduction of the left pisiform under general anesthesia with tourniquet control. A volar longitudinal skin incision was made along the FCU tendon just proximal to the wrist crease, extending distally. The ulnar nerve was identified, and the release of Guyon’s canal was performed. The ulnar nerve was in continuity and not found to be confused. The FCU tendon was split longitudinally, and the dislocated pisiform bone was identified. Bone anchor suture 2.4 mm was inserted over the distal pole of the pisiform, and suture imbrication of the split FCU tendon was performed to reduce and keep in place the dislocated pisiform. The wrist was immobilized in a neutral position for three weeks (Figure 4) followed by 30° in wrist extension for another three weeks. 

**Figure 5:**
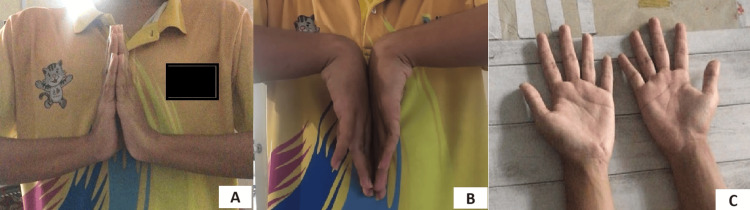
Clinical functional recovery of left wrist at four years postsurgery. Clinical photographs of (A) wrist dorsiflexion, (B) palmar flexion, and (C) resolution of left wrist deformity.

Postoperatively, the patient made an uneventful recovery. At two weeks postoperatively, there was a complete resolution of his pain and numbness. He was started on protected physiotherapy. By the following two months, the patient had full recovery of his grip strength and had returned to normal school activities. Plain radiographs at two months revealed the pisiform bone to have maintained reduction. To date, there have been no instances of re-dislocation, and the patient has been symptom-free for the past four years (Figure 5).

## Discussion

The pisiform bone is sesamoid, found positioned within the FCU tendon, like the patellar bone suspended by the patellar tendon and the quadriceps femoris tendon. The pisiform bone is part of the proximal row of carpal bones of the wrist. It has a flat dorsal surface, which articulates with the triquetrum and forms the pisotriquetral (PT) joint [1]. The volar aspect of the pisiform bone is attached to the FCU tendon.

There are 10 soft tissue attachments to the pisiform bone, as described by Pevny et al. They included the FCU tendon, extensor retinaculum, abductor digiti minimi, transverse carpal ligament, anterior carpal ligament, ulnar collateral ligament, triangular fibrocartilage complex, pisohamate ligament, pisometacarpal ligament, and PT joint fibrous capsule [2].

Stabilization of the pisiform bone is primarily by the FCU tendon, ulnar PT ligament, pisometacarpal, and pisohamate ligaments [2-4]. The FCU tendon is the only dynamic structure that acts directly on the pisiform [4]. Contraction of the FCU tendon during normal wrist flexion pulls the pisiform bone in a proximal direction.

Two primary mechanisms underpin acute pisiform dislocations [5,6]. The first involves direct external forces applied to the pisiform bone. The second mechanism is due to FCU contraction forces on the pisiform. This scenario can be seen in cases of a fall on an outstretched hand whereby the dorsiflexion of the wrist coupled with a strong forceful FCU contraction on impact leads to proximal displacement of the pisiform [4,7]. The pisiform can also become dislocated when one flexes the wrist while lifting heavy objects due to the forceful contraction of the FCU tendon pulling the pisiform proximally [8]. In our case, the mechanism of injury was not clear as the patient denies any trauma to the wrist. However, the likely mechanism of dislocation was from strong FCU tendon contraction, explaining the pisiform bone dislocation in a proximal direction as evident on lateral plain radiographs. 

Diagnosing pisiform dislocation hinges on good history-taking to determine the mechanism of injury. Additionally, clinical assessment should be conducted to evaluate the presence of pain, swelling, and deformity in patients who present with ulnar-sided wrist pain. This is followed by further investigations such as plain radiographs. Admittedly, plain radiographs may not always reveal the dislocation in standard views, necessitating oblique radiographs and radiographs of the contralateral wrist for comparison. A study by Jameson et al., assessing the PT joint and pisiform in motion, suggested at least three semi-lateral anteroposterior views of the wrist [9]. These X-ray views were obtained with the wrist in a neutral position with 30° supination, the wrist in full extension with the forearm in 30° supination, and active and passive wrist flexion with 45° forearm supination while the thumb is fully abducted [4,9]. Nevertheless, confirmation of the dislocation can be evident on CT scan images. Additionally, magnetic resonance imaging can provide insight into associated ligamentous and soft tissue injuries.

Management of pisiform dislocations can either be nonsurgical or surgical. The treatment option for the nonsurgical route includes closed manipulative reduction by repositioning the dislocated pisiform and immobilizing it in a cast. This can be attempted for acute dislocations [4,8,10]. Immobilization strategies following closed reduction vary because the stability of the pisiform is dependent on the wrist position in which it is immobilized [1]. Sundaram et al. advocated for immobilization of the wrist in dorsiflexion and radial deviation, while Sharara and Farrar reported keeping the wrist pronated and flexed to relieve tension in the FCU [8,11]. Campbell and Magi also reported good results in maintaining pisiform reduction by immobilizing the wrist in pronation, flexion, and ulnar deviation [12].

Open reduction becomes necessary if closed reduction fails or when patients present late, and the diagnosis is delayed. As was the case with our patient who did not present acutely and had clinical evidence of ulnar nerve compression. We performed an open reduction to relocate the pisiform bone and imbrication of the split FCU tendon to re-envelop the dislocated pisiform bone back to its original location. In addition, exploration and decompression of the Guyon’s canal were performed given the ulnar nerve compressive symptoms experienced by the patient.

Other authors advocate for pisiform bone excision as an alternative surgical treatment [1,7,12-13]. This option is considered in situations where there is recurrent pisiform dislocation causing persistent pain and the development of PT arthritis. Minami et al. and Ishizuki et al. reported excision of the pisiform bone with no residual disability in their case reports [1,7,14]. Resection of the pisiform bone was not considered in our patient, given the patient’s young age and because this was the first episode of dislocation.

The pisiform's unique anatomy and role in wrist stability make isolated dislocations intriguing yet challenging to manage. The rarity of this condition contributes to diagnostic delays and underscores the importance of maintaining a high index of suspicion in patients who present with ulnar-sided wrist pain and deformity. Any missed pisiform dislocations can lead to persistent wrist pain and complications such as PT arthritis. It is also important to screen and observe for contralateral pisiform involvement in cases with atraumatic pisiform dislocation.

## Conclusions

As more cases are reported and treatment strategies refined, a comprehensive understanding of this uncommon condition will aid in providing timely referrals to orthopedics specialists and hand surgeons to provide effective and appropriate treatment. 
